# Solutions in radiology services management: a literature review*

**DOI:** 10.1590/0100-3984.2014.0065

**Published:** 2015

**Authors:** Aline Garcia Pereira, Lizandra Garcia Lupi Vergara, Eugenio Andrés Díaz Merino, Adriano Wagner

**Affiliations:** 1 Fellow Master degree in Production Engineering, Program of Post-graduation in Production Engineering – Universidade Federal de Santa Catarina (PPGEP-UFSC), Florianópolis, SC, Brazil.; 2 PhD, Professor, Program of Post-graduation in Production Engineering – Universidade Federal de Santa Catarina (PPGEP-UFSC), Florianópolis, SC, Brazil.; 3 Professor, Instituto Federal Farroupilha, Fellow PhD degree, Program of Postgraduation in Production Engineering – Universidade Federal de Santa Catarina (PPGEPUFSC), Florianópolis, SC, Brazil.

**Keywords:** Radiology, Management, Solution

## Abstract

**Objective:**

The present study was aimed at reviewing the literature to identify solutions for problems observed in radiology services.

**Materials and Methods:**

Basic, qualitative, exploratory literature review at Scopus and SciELO databases, utilizing the Mendeley and Illustrator CC Adobe softwares.

**Results:**

In the databases, 565 papers – 120 out of them, pdf free – were identified. Problems observed in the radiology sector are related to procedures scheduling, humanization, lack of training, poor knowledge and use of management techniques, and interaction with users. The design management provides the services with interesting solutions such as Benchmarking, CRM, Lean Approach, ServiceBlueprinting, continued education, among others.

**Conclusion:**

Literature review is an important tool to identify problems and respective solutions. However, considering the small number of studies approaching management of radiology services, this is a great field of research for the development of deeper studies.

## Abstract (pt)

**Objetivo:**

O objetivo deste trabalho é levantar e identificar, na literatura, soluções no âmbito da gestão para os problemas encontrados na área de radiologia.

**Materiais e Métodos:**

A pesquisa é um estudo bibliográfico, de natureza básica, de abordagem qualitativa, do tipo exploratório. Foram utilizadas as bases de dados Scopus e SciELO e os *softwares* Mendeley e Illustrator CC Adobe.

**Resultados:**

Nas bases de dados foram contabilizados, ao todo, 565 documentos, dos quais 120 tinham pdf *free*. Entre os problemas do setor estão: agendamento de horários, humanização, falta de treinamentos, pouco conhecimento e utilização das técnicas de gestão e interação com o usuário. A gestão de *design* apresenta soluções interessantes para o serviço, tais como *Benchmarking, CRM, Lean Approach, ServiceBlueprinting*, educação permanente, entre outras.

**Conclusão:**

Na busca de levantamento de problemas e soluções, a revisão de literatura é uma importante ferramenta, porém, tendo em vista os poucos estudos encontrados no âmbito de gestão na área, é importante realizar estudos mais profundos, o que torna a área um vasto campo a ser estudado.

## INTRODUCTION

After the discovery of X- rays by Roentgen (1895), radiology developed as a medical specialty^(1)^. In 1897 the Belgian Government suggested that all hospitals should be equipped with X-ray apparatuses, evidencing the importance of the method for radiodiagnosis^(2,3)^. A radiology service can comprise methods relying both on ionizing and non-ionizing radiations, covering conventional radiology, fluoroscopy, nuclear medicine, computed tomography, mammography, interventional radiology, bone densitometry, ultrasonography and magnetic resonance imaging, among others^(4)^.

In order to achieve a satisfactory performance, the radiology service must be properly managed. However, one observes that in Brazil management techniques are poorly utilized in the health area^(5)^. Among other, problems encountered in the area include: over-crowded radiology departments; problems in service scheduling (due to tardiness of patients and physicians)^(6-8)^; lack of humanization of the professionals; focus on demand rather than managerial aspects; lack of knowledge on applicable Brazilian law in the field^(9)^; diagnostic errors^(10)^; interaction between the health industry and users in the sphere of the social media^(11)^.

With such problems in mind, the following question emerges: what are the possible solutions for management problems observed in radiology?

The focus on such a theme was motivated by the problems observed in the field, as well as by reports from professionals in the area approaching the need for training on image acquisition and processing, radioprotection, biosafety, humanization, quality control and new technologies^(9,12,13)^. Additionally, as reported by Tizon^(9)^, there is scarce literature on the management of radiology services.

The present article is a literature review developed in the period from March thru May of 2014, on the possible management solutions for problems observed in radiology services. In that sense, a search was carried out in the Scopus and SciELO databases, as well as in other media, such as books, regulations, theses, among others. The dates of publication of the utilized documents were between 1982 and 2014.

The present study was aimed at locating and identifying in the literature, management solutions for problems encountered in the field of radiology.

## MATERIALS AND METHODS

The present article is a literature review aimed at locating and identifying management solutions for problems observed in the field of radiology.

With respect to the nature of the study, it comprises a basic investigation, as it is aimed at generating useful knowledge for scientific development, and not for practical application purposes^(14)^.

The problem is qualitatively approached, as the investigation focuses on the "realm of meanings, reasons, aspirations, beliefs, values and attitudes". The approach is also descriptive, as the investigators tend to analyze data in an inductive way^(15)^.

From its objectives' point of view, the present study is exploratory, with the following objectives: "*to develop hypotheses; increase investigators' awareness of a given environment, fact or phenomenon, in order to further and more accurately investigate in the future, or to modify and clarify concepts*"^(14)^.

As regards technical procedures, the investigation is a literature review. A literature review is defined as an investigation where previously published investigations are synthesized, generating conclusions on the theme of interest^(16)^. The purpose of such an investigation is to "*place the investigator in direct contact with everything that has been written, said or filmed about a given subject*"^(14)^. Thus "*it is not a mere repetition of what has been previously said or written [...], but provides an analysis of a theme under a new focus or approach reaching innovative conclusions*"^(14)^.

According to Ferrari^(17)^, a possible script for a literature review includes:

- Surveying the publications on the subject at libraries.
- Selection of reference sources (indices, bibliographies, abstracts, progress, yearbooks).
- Reference to technical-scientific dictionaries.
- Personal consultations on the subject with experts and specialists.
- Bibliographic investigation itself.

The investigation was carried out in two phases. The first one consisted in searches at databases. At the Scopus database, one utilized signs and Boolean operators with the following descriptors: *radiology; service management; interventional radiology and service design*. At the SciELO database, the descriptors were: *radiologia (radiology); design; gestão (management); ferramentas de gestão (management tools)*; and *radiologia intervencionista (interventional radiology)*. After reading the titles, the abstracts related to the purpose of the present study were selected and read. Then, the search for pdf free files was carried out, and the texts were fully read and those which would be utilized in the present study were selected. The descriptors as well as the number of documents found in this phase are described in Table 1.

**Table 1:** Descriptors utilized at the Scopus and SciELO databases.

Descriptors	Number of documents found	Selection by title	Selection by abstract	Pdf free	Final selection
("radiology") AND ("service management")	307	150	80	60	7
("interventional radiology") AND ("service management")	12	7	7	7	1
("service design") AND ("radiology")	50	21	19	19	0
("service design") AND ("interventional radiology")	6	4	3	3	0
Radiologia / Radiology	151	42	20	20	7
Radiologia e *design* / Radiology and design	17	1	1	3	1
Radiologia e gestão / Radiology and management	4	2	2	2	0
Ferramentas de gestão / Management tools	1	1	1	1	0
Radiologia intervencionista / Interventional radiology	17	5	5	5	2

In the second phase, for a better theoretical grounding, a survey was carried out in books, courseworks, dissertations, theses, standards and regulations, as well as searches at websites of companies that provide services in the field, among them, the Michigan University site^(4)^, which has been providing services in the field of radiology for more than 100 years, and also the site of Colégio Brasileiro de Radiologia e Diagnóstico por Imagem (CBR) (Brazilian College of Radiology and Imaging Diagnosis)^(18)^, a Brazilian entity that represents physicians of the radiology and imaging specialties, founded in 1948.

The open Mendeley software was utilized as a tool for reading and managing the scientific articles, papers and pdf files, and the Illustrator CC Adobe was utilized as a design tool.

## RESULTS

The design of services emerged with the mission of applying concepts and practices for the creation of services and internal processes more appropriate to the people's reality^(19)^. It may be understood as an area "*focused on the project and on the people as human beings which contribute with the co-creation of the enterprise's value, and not only as users of a service*"^(20)^. The client/service interface is the most relevant point of interaction in the service process, as it is at that particular item that the service's image and identity materialize^(21)^. According to Mager^(22)^, the holistic approach is among the basic principles of service design, considering services as living systems that have the users as active partners in the creation of value.

In the field of radiology, such a holistic view focused on the human being is important, as in most of the problems, it is the fundamental feature for management in conjunction with its relationship with the environment and the technologies.

The solutions for problems found in the documents selected for the study are described along the present article.

### Waiting time and problems with appointments scheduling

In order to solve problems related to waiting time as well as over-crowded radiology services and problems with appointment times (caused by tardiness of patients or physicians), some authors^(6-8)^ suggest focusing on three items regarding the appointments scheduling:

- Naming rule - One should know the environment and study the probability of non-attendance, the variability of service times, number of patients and time required for the performance of the procedure.
- Patients' classification - Make up the schedules with basis on the patients' characteristics and consultation times. Example: is the patient a new patient or a returning patient? What is the procedure the patient will be submitted to (what is the time required for that procedure)? What is the patient's age range? Does the patient present with any disablement that might influence exam time?
- Schedule adjustments (due to non-attendance) - As patient absenteeism is a reality, a suggestion to the services is to contact the patients in advance in order to confirm the appointment, and in case the appointment is not confirmed, the service should call other patients in the waiting list to have their exams made. Aiming at a better management of the waiting list, one suggests having at such a list the names, phone numbers as well as preferred times of other patients.

The service and its staff should keep in mind the punctuality in rendering the services, as well as accuracy in the procedure performance in order to avoid the need for repetitions, thus avoiding waste of time and money.

### Interaction with users

With the advent of new technologies, the interaction between enterprises and people has gone through changes. The social media have changed the relationship with the world, modernizing the means of acquiring information. According to Castro^(23)^, there is a new work organization based on collaboration, sharing and information access.

The social media are not only an entertainment means and have actually become an important communication and relationship channel between enterprises and the people^(23)^. A solution to improve the interaction between radiology clinics/hospitals and users is the use of such media.

A study developed by Huang et al.^(11)^ observed 23,300 posts at social networks such as Facebook and Twitter during December/2012 from 172 North American hospitals. One observed that the interaction through the social media is effective for knowing the visitors. The social media operates as a two-way communications channel, where the enterprise's marketing occurs while visitors provide feedback, and it is possible to listen and know the users. At Facebook, 65% of the users commented on the posts from the hospitals, with 15% sharing thoughts and emotions; on the other hand, at Twitter, 82% of the posts were responses, with 8% sharing thoughts and emotions online.

Currently, it is estimated that Facebook has approximately 800 million users. In August/2014, CBR^(18)^ launched its official page at that network with the purpose of obtaining visibility for its activities, guidance and news, besides continuing with its efforts towards approaching the lay audience. In one month, the profile received 490 likes, while providing important information on national and international congresses, management courses, programs of accreditation in imaging diagnosis, reports on health and radiology, among other subjects.

Another option for improvements in the interaction with radiology service users is Customer Relationship Management (CRM). Such a customer relationship management tool requires managers to monitor critical issues affecting customer satisfaction and fidelity, as well as those which potentially increase enterprise's income while enhancing competitive advantage^(24)^.

As observed, in the interaction between the client and the service, the client's opinion is an important tool for the development of innovations in the sector. Learning client's opinions is a method for identifying user needs, thus identifying opportunities to improve service development and quality of rendered services^(24)^. In radiology, such a knowledge is very important to support decisions on investments and priorities at the services.

### Equipment and tests

The radiology apparatuses' technology is constantly evolving^(25)^. In interventional radiology, for example, they are usually highly sophisticated^(1)^ and should be kept in good working conditions, as a simple problem such as an improperly functioning collimator can generate errors such as imaging artifacts and additional exposure to ionizing radiation for both the patient and staff.

Considering that the diagnostic image is one of the main points in the decision making, it is of utmost importance that the imaging and image acquisition systems be kept in good working conditions, providing the best quality images with the lowest possible exposure to radiation^(26)^.

There are various tests to which the apparatuses must be submitted at different periodicities, according to Ordinance 453/98^(27)^. As regards tests that are not required by Brazilian regulations, services in the country may use international regulations, as Brazil is a member of the United Nations Organization.

Forms developed by the Health Surveillance Directory of Santa Catarina^(28)^ represent a suggestion for inspections in the radiology area.

### Multidisciplinary knowledge

Radiology teams are multidisciplinary by their nature, comprising radiology technicians and/or technologists, and, frequently, nurses, nursing technicians, cardiologists, orthopedists, vascular surgeons, neurologists and many other professionals who, many times, are not properly trained in radiological protection^(29)^. With a view on the increasing technological complexity, continued education is a key point to be implemented in the sector, as learning and teaching are a part of the daily work in the organizations^(12,30)^.

One of the main problems observed in the field is the poor or even lacking knowledge on what ionizing radiation is, both from the part of the services staff and users^(9,31)^. Possible solutions which management can resort are:

- Capacitation for personnel development - Possible topics include: management; radioprotection; humanization (treating people courteously), imaging acquisition and processing; and biosafety.
  Radioprotection is a set of measures aimed at protecting human beings and their descendants, as well as the environment, from deleterious effects caused by exposure to ionizing radiations. Among its basic principles, three should be highlighted: doses justification, optimization and limitation. As regards legal principles which establish quality control testing requirements in radiology, one should highlight those established by Ordinances 453/98^(27)^ and CNEN NN3.01^(32)^. In the management of a radiology service, it is necessary to comply with the current Brazilian standards, among those, item 3.38 of Ordinance 453/98, which establishes the implementation of yearly training programs^(27)^. Such programs should approach equipment's operation procedures; utilization of radiological protection clothing (RPC) for patients, staff and companions; utilization of dosimeters; patients and staff exposure to ionizing radiation, local hygiene (biological risks); as well as other aspects involved in the appropriate procedures performance.
  Considering that exposure to ionizing radiation^(26,33)^ can cause stochastic and/or deterministic effects, the professionals should have the knowledge on doses and undergo monthly monitoring, as well as utilize RPCs. Wearing RPCs theoretically reduces absorbed dose by 86-99%, with reduction of up to 88% being observed in practice for patients, and 90% for occupationally exposed individuals during an interventional procedure^(33)^. Unfortunately, one observes that in some sectors, the RPCs are in a very poor condition, thus the staff is physically exposed to the loads of radiation^(12)^.
  As regards the premises, they should be in good conditions (clean environment, with easily cleanable surfaces)^(1)^. The professionals should be aware of the working environment risks in order to minimize them.
  The utilization of new technologies in the sector, as in the case of information and imaging systems (DICOM, PACS, RIS, HIS, among others), should be gradually implemented in order to allow for an appropriate adaptation of professionals^(34)^.
- Booklets and flyers - With a view on the lack of knowledge on ionizing radiations by many (professional and patients), the services may develop and print explanatory booklets and flyers relying on simple means of conveying the necessary information. A good example is the work developed by Instituto de Radioproteção e Dosimetria^(31)^, aimed at drawing attention to the effects caused by the interaction between radiation and the organism, providing radiological protection notions by means of posters, booklets and comic books.
- Diagnostic errors - According to Itri et al.^(10)^, some mistakes are made by radiologists during diagnosis. One way to minimize errors is to have reports reviewed by more than one individual (peer reviews). Other suggestions include: implementation of follow-up programs for radiology residents, particularly regarding reporting, and further studies on radiology information, image filing and communication systems^(35)^, for optimization of reports.

### Management

In order to improve operational efficiency in the sector, Kruskal et al.^(36)^ have developed a study at a radiology department, utilizing the Lean method, and demonstrated some models for process standardization and improvement in processes order flow. Solutions include: utilization of check-lists, flowcharts and the "5S" principle; the utilization of notices and reminders (visual signs) in the sector increases safety and helps minimize human errors.

A useful tool for those willing to improve their services is Benchmarking, which consists of analyzing and comparing the service with others^(5)^, also an excellent method to be adopted by those starting up a service; the management processes help the enterprise to incorporate changes, develop new techniques and introduce innovations. The service may observe and know other neighboring services or even foreign ones, such as the centenary Michigan Radiology Department^(4)^. A valuable guiding principle for an enterprise is the conceptualization of its mission, vision and values.

Management courses like those offered by the CBR program represent an important tool for administrators and physicians^(37)^. They are given by specialists in the market of diagnostic medicine, with an education in medicine, or by executives from the sector; it is divided into modules and approaches commercial management, financial management and quality certification among other topics.

**Figure 1:**
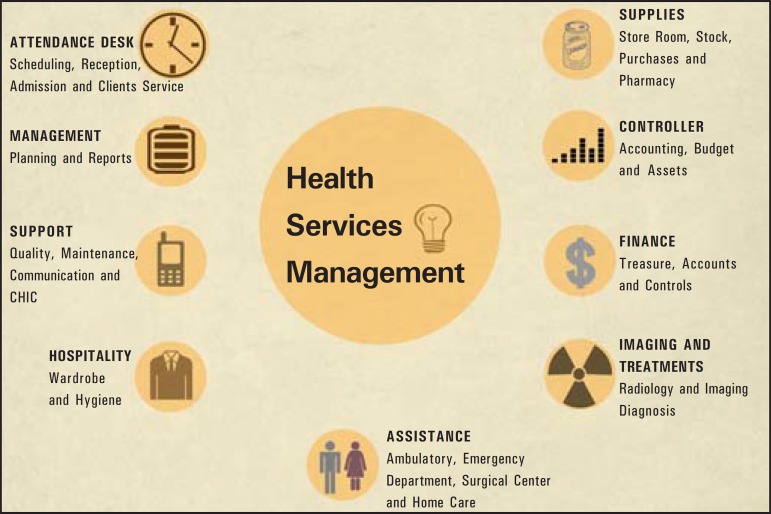
Coverage areas of a management system in the health sector.

As a support in the management of administrative and assistance activities, the service can utilize a health management system, which should approach different areas such as reception, assistance, management, hospitality, support, controlling and supplies, among others (Figure 1). An example is Tasy^(38)^, a patient-focused system.

Among other suggestions for improvements in the sector is the utilization of the ServiceBlueprinting, a management model for analysis, visualization and optimization of service processes, as well as implementation of human resources management^(39)^, and utilization of the ABC (activity-based costing) method for analyzing the service costs^(25)^.

## DISCUSSION

Management can be understood as a "*set of efforts aimed at planning; organizing; directing or leading; coordinating and controlling the activities of individuals who associate in order to achieve a common result*"^(40)^. It is very important for the organization, as it contributes to the structuring of meanings in order to adapt people to the surrounding world, facilitating interpersonal relationships^(41)^.

In radiology, managers must have a comprehensive vision, knowing the organizational culture as well as the internal and external environments, thus allowing for a systematic and interactive process, which is, therefore, an interdisciplinary process^(42)^. With a view on the problems observed in the service, managers can follow some steps before moving towards solutions, such as: a) understand the situation; b) describe the situation, observing the potentialities and weaknesses; c) list and demonstrate the improvement conditions (solutions); d) and finally, provide an opinion or judgement.

**Figure 2:**
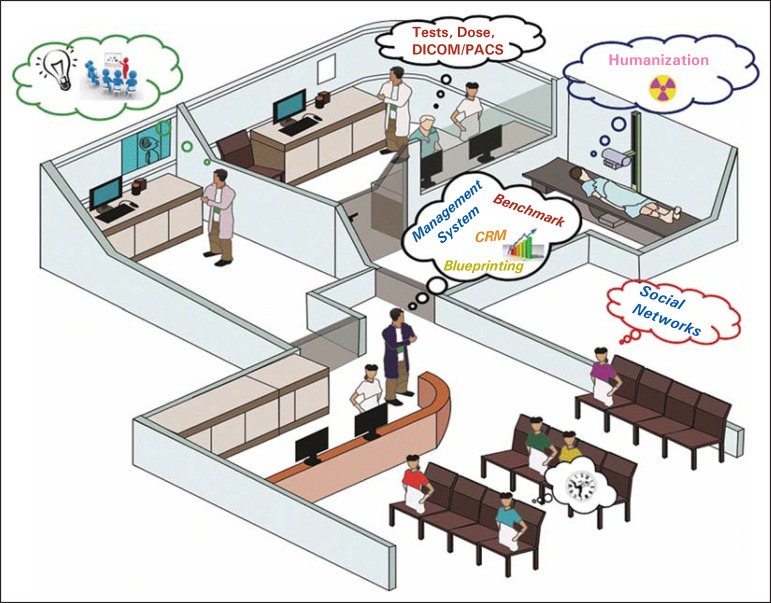
Radiology service. (Credits: Felipe Coelho Maestri – graduate student of Design at UFSC, Adobe Illustrator CC).

In a general overview of the problems and solutions of a radiology service (Figure 2), one observes that the "knowledge" factor is preponderant, so continued education is highly necessary, as well as capacitation and training, for the development of such professionals^(43,44)^. On the other hand, the utilization of booklets^(31)^ represents a valuable instrument for the dissemination of knowledge among the service users.

Another important factor to be considered is the interaction between the enterprise the service belongs to and the user. In a study developed by Silva et al.^(45)^, among the reasons why users return to a radiology service, the following were highlighted: trust and competence of the health professionals, scheduling flexibility and results reliability. Such factors reinforce the need for permanent education at the service, efforts towards errors reduction, and a good scheduling timetable. As the indispensable improvements were considered, the following aspects were highlighted: waiting time for the performance of the exam and the dimensions of the waiting room.

Other items that should be constantly observed and improved are: image quality; selection of technical parameters (kV, mA, time) to minimize acquisition time and exposure dose (both to patients and staff); utilization of RPC (thyroid shields, lead aprons, goggles, gloves, etc.) as well as other barriers (petticoats and eye protection); integrity of the RPCs and of the barriers, as they greatly reduce the exposure to ionizing radiation^(33)^. Additionally, quality assurance and management programs should be implemented^(46)^.

## CONCLUSION

Radiology services exist for the clients and, for that reason, they should be focused on the users' satisfaction. In the search for improvement opportunities, the service must know the problems and their root causes and, based on such knowledge, develop actions to solve such problems. For this purpose, one of the options is carrying out surveys with clients, external and internal markets (benchmarking) as well as with the service workers.

In the search for problems and solutions, the literature review is an important tool in order to acquire a general overview on the subject. However, because of the small number of articles approaching management in the field of radiology, it is important to undertake *in loco* case studies about radiodiagnosis market, management methods (commercial, financial and human resources), management models and tools in order to obtain more comprehensive data on the subject.

Among the different suggestions for studies to be undertaken, the following can be mentioned: implementation of permanent education in the radiology service; survey on the profiles of management professionals in the service; identification of the tools utilized in the services management; identification of the challenges faced by the management in the service; definition on how interaction occurs between radiology technologists and other professionals in the field; definition on how people are managed in radiodiagnosis services.

Knowledge on the problems in radiology is important and so is finding solutions for such problems. However, more than gathering data, information and tools, it is necessary to know how to implement the changes required to develop the organization.
